# Exosomal Protein Markers as Potential Non-Invasive Biomarkers for Colorectal Cancer

**DOI:** 10.3390/ijms262211060

**Published:** 2025-11-15

**Authors:** Maciej Skrzypek, Danuta Piotrzkowska, Julia Adamkiewicz, Mateusz Prusisz, Michal Bijak, Leslaw Gornik, Lukasz Dziki, Ireneusz Majsterek

**Affiliations:** 1 Department of Clinical Chemistry and Biochemistry, Medical University of Lodz, 92-215 Lodz, Poland; 2 Clinic of General and Oncological Surgery, Medical University of Lodz, 92-215 Lodz, Poland; 3 Biohazard Prevention Center, University of Lodz, 90-236 Lodz, Poland

**Keywords:** colorectal cancer, biomarkers, exosomes, exosome isolation methods, size exclusion chromatography, ultracentrifugation

## Abstract

Exosomes are small extracellular vesicles found in body fluids. They contain proteins and nucleic acids that reflect the condition of the parent cell. Because of this, they may be useful in colorectal cancer (CRC) diagnostics. In this study we compared three plasma isolation methods: ultracentrifugation (UC), size exclusion chromatography (SEC), and a commercial Exo-spin kit. The material obtained with each method was checked for protein content, purity, and vesicle integrity using protein measurements, Western blots, and scanning electron microscopy. From these results SEC was chosen for further use. Exosomes isolated with this method were tested by an enzyme-linked immunosorbent assay (ELISA) for four CRC-related proteins: HSP70, CK19, CA125, and TAG72. Patient samples showed higher levels of HSP70, CK19, and CA125 than controls. TAG72 levels did not differ. In addition, men had more HSP70 than women. These findings show that SEC can be applied for exosome isolation from plasma and that specific proteins detected in exosomes, including HSP70, CK19, and CA125, may serve as promising markers for non-invasive colorectal cancer diagnosis and monitoring.

## 1. Introduction

Exosomes are small extracellular vesicles, typically ranging in size from 30 to 200 nanometers [1,2]. These nanostructures have a rounded shape and are enclosed by a lipid bilayer and specific membrane proteins characteristic of exosomes. Interestingly, the composition of the exosomal membrane often reflects the cell type from which they originate [3]. Exosomes encapsulate a variety of bioactive molecules, including proteins, lipids, and genetic material such as double-stranded DNA, messenger RNA, microRNAs, and long non-coding RNAs [4,5,6]. The content they carry tends to be closely linked to the functional state and identity of the parent cell, making exosomes a valuable source of molecular information, especially in disease-related studies [4,6]. The cargo of exosomes is determined by the parent cell through the protein complex ESCRT (endosomal sorting complex required for transport) [7]. Exosomes are formed by the protrusion of the cell membrane into the middle of the endosome, resulting in the formation of late endosomes [8]. They are subsequently released into the intercellular space by fusion of the late endomembrane with the cell membrane (exocytosis). Exosomes are present in all body fluids, namely blood, saliva, urine, cerebrospinal fluid, and breast milk [9]. Their ability to migrate passively in body fluids enables them to travel long distances in the body and function as transmitters, thereby regulating the function of target cells in physiological and pathological processes [10,11].

Characterization of exosomes commonly relies on the identification of specific protein markers confirming their endosomal origin and purity. The most frequently used positive markers include ALIX, CD81, and CD9, which are involved in exosome biogenesis and membrane organization [1,3,5]. ALIX participates in the ESCRT pathway, which is responsible for intraluminal vesicle formation, whereas CD81 and CD9 are tetraspanins that regulate membrane microdomain architecture and selective cargo sorting [3,7,12,13]. In contrast, albumin and calnexin serve as negative markers. Albumin, a major plasma protein, is secreted via the classical pathway, and its detection indicates contamination with serum components [9,13]. Calnexin, an endoplasmic reticulum (ER) membrane chaperone, is unrelated to endosomal structures and is recommended as a negative marker by the MISEV2018 guidelines [13]. The combined presence of ALIX, CD81, and CD9 together with the absence of albumin and calnexin confirms the endosomal origin and high purity of isolated exosomes [13].

Cancer cells have been shown to secrete exosomes with molecular compositions distinct from those of normal cells [4,12,14]. Exosomal microRNAs have recently emerged as potential biomarkers for colorectal cancer (CRC), with RNA sequencing studies showing distinct miRNA profiles between patients with cancer and healthy individuals [15]. In addition to RNA, exosomes may also contain tumor-associated proteins such as CK19, HSP70, CA125, and TAG72. Detecting them in blood plasma opens the possibility of using exosomes as a non-invasive diagnostic tool [16]. Some markers showed promising diagnostic value, although not all reached statistical significance. Still, exosomal content may serve as a basis for early detection and disease monitoring in the future [17]. Several studies have demonstrated that both the quantity and molecular composition of circulating exosomes correlate with disease stage and tumor progression in CRC. Tumor cells release increased numbers of exosomes compared with normal cells, and the protein, as well as the RNA cargo, of these vesicles changes dynamically during tumor growth, invasion, and metastasis [4,14,15,17,18]. Such alterations reflect ongoing cellular reprogramming and therapy-induced responses, making exosomes promising markers not only for early diagnosis but also for longitudinal monitoring of treatment efficacy and disease recurrence. Therefore, analysis of exosome-derived biomarkers offers a minimally invasive approach to track CRC progression and assess patient responses in real time [4,15,17,18]. Beyond diagnostics, exosomes are being actively investigated for therapeutic applications. Their potential has been explored not only in cancer treatment but also in conditions such as neurodegenerative, cardiovascular, and respiratory diseases [18,19]. Innovative strategies, including engineering the exosomal content, have been developed to enhance their therapeutic efficacy, establishing a versatile platform for drug delivery and gene therapy [20].

The biggest problem for further research into the contents, functions, and uses of exosomes is their effective separation from body fluids and culture media [21]. Currently, exosome isolation relies on methods such as size exclusion chromatography (SEC), ultracentrifugation (UC), density gradient centrifugation, ultrafiltration, diafiltration, immunoaffinity, and organic solvent precipitation [22]. In our study, we focused on comparing three methods for isolating exosomes, namely UC, SEC, and a commercially available method for isolating exosomes using an Exo-spin column.

UC relies on physical differences between particles, such as size, shape, mass, and density, and uses centrifugal force to separate them. The specific conditions, such as spin duration or speed, often depend on what type of material is being processed. In practice, the number of centrifugation steps and the type of rotor used can vary from case to case [23,24]. The larger and heavier the particles, the easier they are to separate from the solution [25]. Smaller particles require repeated centrifugation at forces tens to hundreds of thousands of times greater than Earth’s gravity to achieve their isolation [26]. For serum samples, dilution of the starting material and additional filtration steps are also necessary.

SEC allows separation based on particle size, rather than mass [27]. The column is packed with a stationary phase consisting of a porous polymer (Sephadex, Sepharose, Biogel P, and Sephacryl) [28,29], while the mobile phase is a liquid that elutes the molecules. The separation of the particles occurs under the influence of gravity and depends on their size. Smaller particles penetrate the pores of the gel beads that constitute the stationary bed, whereas particles larger than the bed pores do not penetrate them and elute first [30]. The penetration of smaller particles into the pores increases their residence time, resulting in later elution. The type of particles separated from solution can be controlled by selecting a bed with an appropriate pore size.

Among the methods applied for exosomal protein analysis, enzyme linked immunosorbent assay (ELISA) is one of the most frequently used. The assay enables sensitive and quantitative detection of markers such as CK19, HSP70, CA125, and TAG72 in plasma-derived exosomes. Because it is relatively simple, reproducible, and already familiar in clinical laboratories, ELISA serves as a practical tool to complement molecular techniques and strengthens the potential of exosomes as non-invasive biomarkers [31].

Given their molecular composition, role in extracellular communication and accessibility in body fluids, exosomes constitute promising biomarkers for many diseases, including cancer. Their biological, diagnostic and therapeutic potential provides the rationale for the present study.

We hypothesize that the protein markers CK19, HSP70, CA125, and TAG72 present in plasma-derived exosomes can serve as reliable biomarkers for non-invasive diagnosis and monitoring of CRC. Furthermore, we hypothesize that the method of exosome isolation significantly influences their yield, purity, and preservation of molecular content, thereby determining the quality and reliability of the diagnostic data obtained.

## 2. Results

### 2.1. Protein Concentrations in Exosomes Preparations

The bicinchoninic acid assay (BCA) results were used to preliminarily evaluate the protein content of the exosome preparations and enabled continuation of the study using the Western Blot technique (Table 1). All tests used material from the same patient. The specimens were frozen and thawed immediately before the procedure.

BCA test results confirmed the presence of proteins in each preparation. The preparations obtained using the developed isolation methods contained high protein levels, indicating efficient exosome extraction.

### 2.2. Evaluation of Exosome Extraction and Purity

**Figure 1:**
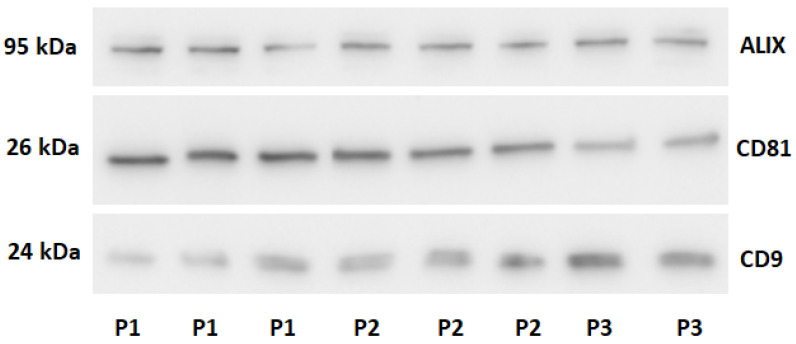
Western blot analysis of preparations for exosomal proteins. P1-ultracentrifugation sample. P2-size exclusion chromatography sample. P3-Commercial kit sample.

**Figure 2:**
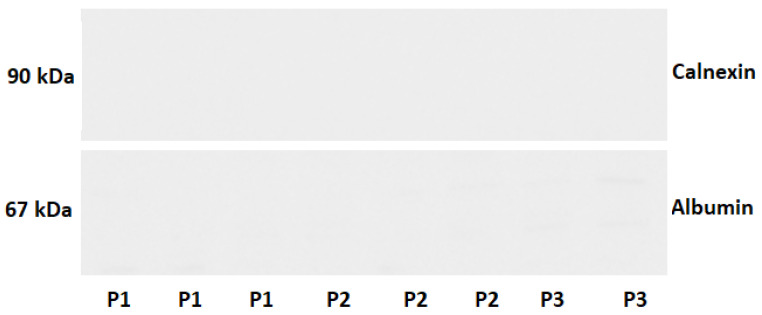
Western blot analysis of preparations for non-exosomal proteins. P1-ultracentrifugation sample. P2-size exclusion chromatography sample. P3-Commercial kit sample.

The Western Blot method technique was used to assess the presence of exosome-associated proteins (ALIX, CD9, CD81) (Figure 1) and non-exosomal proteins (Albumin, Calnexin) (Figure 2), which are plasma-derived contaminants, in the isolated preparations.

The results confirmed the presence of exosomes in samples obtained using each isolation method. Signals for exosome-associated proteins (ALIX, CD81, CD9) were consistently detected across all assays. In contrast, signals for non-exosomal proteins were very weak, indicating the high purity of the preparations and the effectiveness of the developed separation methods.

### 2.3. Scanning Electron Microscopy Analysis of Isolated Exosomes

**Figure 3:**
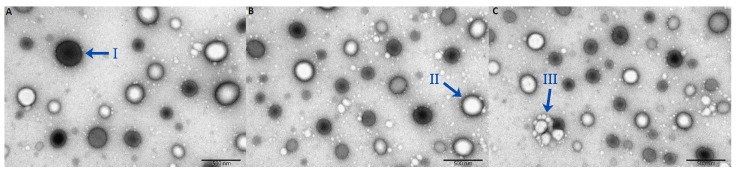
Scanning electron microscope images of plasma-derived exosomes. (**A**) Ultracentrifugation sample; (**B**) Size exclusion chromatography sample; (**C**) Commercial kit sample; (I) Extracellular vesicles; (II) Exosome; (III) Cell debris.

Scanning electron micrographs of the isolated exosomes enabled qualitative assessment of the separation. According to accepted criteria, exosomes range from 30 to 200 nm. The images confirmed the presence of exosomes, other extracellular vesicles, and cell debris in plasma (Figure 3).

Fewer vesicles were observed in samples obtained by UC compared with the other methods, and these vesicles were larger in size. Vesicles in the SEC and commercial kit samples were comparable in both size and number. Across all samples, vesicle diameters ranged from 30 to 200 nm, consistent with the presence of exosomes. The accompanying micrographs showed no evidence of contamination, such as cell debris, thereby confirming the purity of the preparations.

### 2.4. Quantitative Analysis of Exosomal Markers of Colorectal Cancer

ELISAs were used to quantitatively evaluate proteins commonly associated with CRC derived exosomes, including HSP70, CK19, CA125, and TAG72. Exosomes were isolated using SEC.

**Figure 4:**
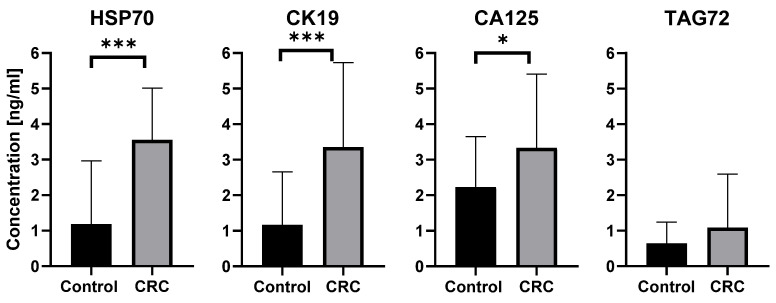
Quantitative detection of exosomal proteins characteristic of colorectal cancer, performed using the ELISA method. Results are presented as mean concentration [ng/mL] ± SD, * *p* < 0.05, *** *p <* 0.0001. CRC-Colorectal Cancer.

ELISA analysis demonstrated that exosomal proteins associated with CRC were detectable in patients’ plasma samples. As shown in Figure 4, the concentrations of HSP70, CK19, and CA125 were significantly higher in CRC patients compared with healthy controls (HSP70 *p* < 0.0001; CK19 *p* < 0.0001; CA125 *p* = 0.0124), whereas TAG72 (*p* = 0.1559) levels did not differ between groups. Data are presented as mean concentration [ng/mL] ± SD.

**Figure 5:**
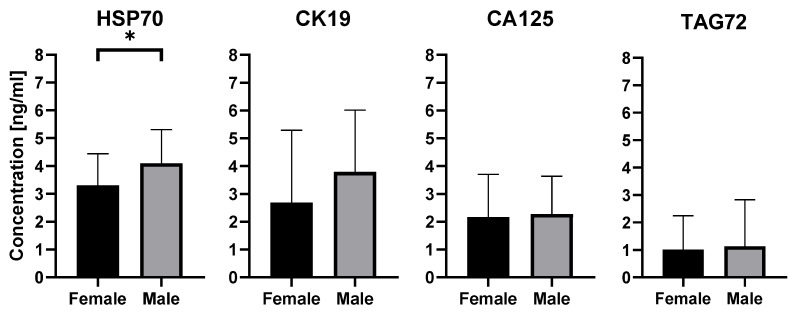
Quantitative detection of exosomal proteins characteristic for colorectal cancer in male and female, performed by ELISA. Results are presented as mean concentration [ng/mL] ± SD, * *p* < 0.05.

Further analysis revealed significant sex-related differences. As shown in Figure 5, men had higher levels of HSP70 than women, and this difference reached statistical significance (*p* = 0.0475).To evaluate the effect of age, participants were divided into two groups: below 60 years and 60 years or older. In the comparison between women and men under 60 years, no significant differences were observed (*p* = 0.7914). In the group aged 60 years and above, statistical significance (*p* = 0.0956) was not reached, although a trend toward higher HSP70 levels in men was noted. For CK19, CA125, and TAG72, no statistically significant differences were observed in relation to either sex or age. Data are presented as mean concentration [ng/mL] ± SD.

## 3. Discussion

CRC remains one of the leading causes of cancer-related morbidity and mortality worldwide [32]. Early detection is critical, as patient outcomes strongly depend on the stage at which the disease is diagnosed [33]. Standard diagnostic tools, such as colonoscopy, biopsy, and imaging, are effective but invasive and costly, which limits their use in routine population screening. This underlines the need for non-invasive biomarkers that could complement existing approaches and improve both the accuracy and accessibility of CRC detection [34].

Exosomes have recently attracted attention as promising candidates in this context. They circulate in all body fluids and carry proteins and nucleic acids that reflect the state of the tumor of origin [13]. Their stability in the bloodstream and accessibility through liquid biopsy make them particularly attractive for diagnostic applications [35]. Several exosome-associated proteins, including HSP70, CK19, CA125, and TAG72, have been linked to CRC in earlier studies [16,36]. Building on this evidence, our study aimed to evaluate laboratory-suitable methods for exosome isolation and to determine the diagnostic utility of these selected protein markers in CRC-derived exosomes.

The developed exosome isolation methods proved effective. Both approaches have specific advantages and limitations and require further refinement. Their efficiency and purity were comparable to those achieved with commercially available kits. Moreover, the methods are accessible to other research teams and can be readily adapted to the specific requirements of individual experiments. This study therefore focused on developing efficient and reproducible methods for the isolation of exosomes from human blood and comparing them with a commercial reference kit. The comparison included an evaluation of the purity and molecular content of the isolated vesicles. Two isolation methods, ultracentrifugation (UC) and size exclusion chromatography (SEC), were selected for detailed analysis. UC is characterized by low procedural complexity and minimal reagent requirements. In this study, we developed a UC-based method for exosome isolation, which preferentially recovered larger vesicles. Exosomes are typically defined as 30–200 nm in size [1,2]. However, the obtained preparations consisted mainly of vesicles larger than 100 nm. Comparable findings were reported by Théra C. et al., who suggested that the loss of membrane integrity in a subset of exosomes may explain this shift in size distribution [37]. Another possible explanation is that the centrifugation speed or duration applied in our protocol was insufficient. Coughlan C. et al. demonstrated that increasing centrifugation speed fourfold across two sequential UC improved the recovery of smaller exosomes [38]. Our protocol therefore facilitates the acquisition of larger exosomes, while the use of lower centrifugation speeds increases accessibility by allowing the use of smaller ultracentrifuges. This feature may be of particular importance for research groups implementing exosome isolation in experimental or diagnostic settings.

SEC has emerged as an alternative method for exosome isolation. Compared with UC, SEC offers the significant advantage of yielding exosomes with greater heterogeneity. Similar observations were reported by Baranyai T. et al. [39]. Unlike centrifugation-based approaches, SEC does not subject samples to high centrifugal forces, thereby minimizing vesicle membrane damage and increasing both the yield and diversity of isolated exosomes. Other studies have also emphasized that the milder separation conditions of SEC promote preservation of membrane integrity [40,41]. An additional advantage of SEC is the possibility of isolating distinct exosome fractions during separation, which is of considerable importance for downstream analyses [42]. However, compared with UC, SEC requires greater precision in column preparation, eluent composition, and sample loading, making it more prone to technical variability and reduced reproducibility. Similar conclusions were reached by Yang D. et al. [43]. Moreover, SEC is a low-throughput method, typically yielding smaller amounts of exosomes than UC. Nevertheless, both developed methods were as effective as the commercial isolation kit (Cell Guidance Systems Exo-spin Ex05-20). However, in the subsequent part of the study, we applied the optimized SEC protocol developed in this work, which provided comparable purity to the commercial kit while allowing the processing of larger sample volumes in a shorter time. Because this method ensured gentle separation conditions, high purity, and preserved vesicle integrity despite lower throughput compared to UC, SEC was selected as the isolation method for further analyses.

To evaluate the molecular cargo of the isolated exosomes, ELISAs were performed for several proteins previously associated with CRC. The selected targets were HSP70, CK19, CA125, and TAG72, have all been described as potential exosome-associated markers in earlier studies [44,45,46,47].For instance, Xiao et al. demonstrated that CK19, TAG72, and CA125 are differentially expressed in exosomes from CRC cells depending on metastatic potential and drug resistance profiles [16]. Moreover, HSP70 has been identified as an exosome-associated chaperone involved in immune regulation in CRC, supporting its value as a diagnostic and prognostic biomarker [36].

ELISA results demonstrated significantly higher concentrations of HSP70 (*p* < 0.0001), CK19 (*p* < 0.0001), and CA125 (*p* < 0.05) in exosomes from CRC patients compared with healthy controls. These findings are consistent with previous reports describing overexpression of these proteins in CRC tissues and blood circulation [16,48], supporting their potential utility as diagnostic indicators. In contrast, TAG72 levels did not differ significantly between the groups (*p* > 0.05), suggesting limited value of this marker in the exosomal context. This may be attributed to its low abundance in the exosomal fraction or to the fact that TAG72 is predominantly associated with other extracellular components, such as tumor-derived microvesicles or free plasma proteins, rather than with exosomes.

In addition to differences between patients and controls, variation related to sex was observed. Men had significantly higher concentrations of exosomal HSP70 than women (*p* < 0.05). This may indicate that sex-related biological factors may influence the protein cargo of exosomes. In participants aged 60 and older, the difference did not reach statistical significance (*p* > 0.05), although a trend toward higher HSP70 levels in men was noted, which may be explained by the decline of androgens with age influencing stress protein expression in tumor and stromal cells and thereby shaping the exosomal cargo [49]. This trend suggests a potential interaction between age- and sex-dependent hormonal regulation and exosomal protein packaging, which warrants validation in larger, longitudinal cohorts.

ELISA-based quantification confirmed that exosomes isolated by SEC retained functionally relevant protein content and could be reliably analyzed for biomarker discovery. These findings support the applicability of the developed protocol for downstream diagnostic applications. The elevated levels of HSP70, CK19, and CA125 in CRC-derived exosomes suggest their potential value in non-invasive monitoring and detection of CRC via liquid biopsy [44,49]. Collectively, these results highlight the diagnostic relevance of exosomal proteins and support their integration into liquid biopsy-based approaches for early and non-invasive CRC detection.

This study evaluated two methods for exosome isolation and compared them with a commercial kit, thereby validating their use in typical laboratory settings. Exosomal protein analysis was performed to identify markers associated with CRC, linking the isolation process to potential clinical utility. These findings support the utility of exosomes as a source of biomarkers in the diagnosis and monitoring of CRC and point to the direction of further research. Extending the analyses to additional classes of exosomal molecules, including microRNAs, DNA, and lipids, will enable a more comprehensive assessment of their role and significance in clinical practice.

Future research will focus on expanding the current findings by including larger, clinically stratified patient cohorts. Moreover, integrating exosomal protein profiling with other omics approaches such as transcriptomics, metabolomics, and lipidomics further advances the molecular understanding of colorectal cancer and supports the development of more robust exosome-based diagnostic and prognostic tools.

## 4. Materials and Methods

### 4.1. Materials

The study involved 92 participants in total. Fifty-two patients diagnosed (biopsy) with colorectal cancer (CRC) were initially enrolled. Among them, 12 patients were included exclusively in a pilot phase to compare different exosome isolation methods and were not included in the statistical analyses. The remaining 40 CRC patients were included in the main experimental and statistical analyses. The control group consisted of 40 healthy volunteers, selected from among those admitted to the Department of General Surgery and Oncology for other reasons unrelated to chronic inflammation, cancer, or neurodegenerative diseases. Blood samples were collected from both patient groups and the control group. The study was conducted after obtaining approval from the Bioethics Committee (no. RNN/288/21KE), and patients provided informed consent prior to blood collection.

The CRC study group included in the main experiment consisted of 16 women and 24 men; the mean age of women was 61 years, and of men, 61.5 years. The control group consisted of 23 women and 17 men; The average age of women was 62 years, and of men was 65 years (Table 2). Before the experiments began, all participants underwent comprehensive medical examinations. Inclusion criteria for the study included: diagnosis of CRC by biopsy, age over 18 years, and the ability to provide informed consent. Exclusion criteria included: age under 18 years, the presence of other cancers, and the inability to provide informed consent.

Twelve ml of venous blood was collected from each participant in EDTA-coated tubes and stored at 4 °C until processed. All samples were processed within one hour of collection, and hemolysis was assessed before further procedures. The material collected from patients diagnosed with CRC was divided into two experimental groups. In the first group, consisting of 12 patients with CRC, different exosome isolation methods were compared. Each blood sample was divided into three equal portions to test the three isolation techniques on the same donor sample and determine which method yielded the best results in terms of yield and purity. In the second group, consisting of 40 patients with CRC, exosome markers specific to CRC were investigated. Exosomes from 40 CRC patients and 40 healthy controls were isolated using SEC method, then exosomes analyzed by ELISA to measure certain protein markers.

### 4.2. Plasma Isolation

Initial centrifugation was performed to separate cellular components from plasma at 1000× *g* for 15 min at room temperature (OHAUS FRONTIER 5916R, Ohaus Corporation, Parsippany, NJ, USA). The separated plasma was then transferred to new Falcon tubes and centrifuged again at 5600× *g* for 30 min at 4 °C to remove residual cell debris and protein aggregates. The resulting plasma was then passed through a cellulose acetate (CA) syringe filter with a pore size of 0.22 μm (Merck Millipore, Darmstadt, Germany) to remove cell debris. The plasma prepared in this way was aliquoted into 1000 μL Eppendorf tubes and frozen at −20 °C.

### 4.3. Ultracentrifuge

**Figure 6:**
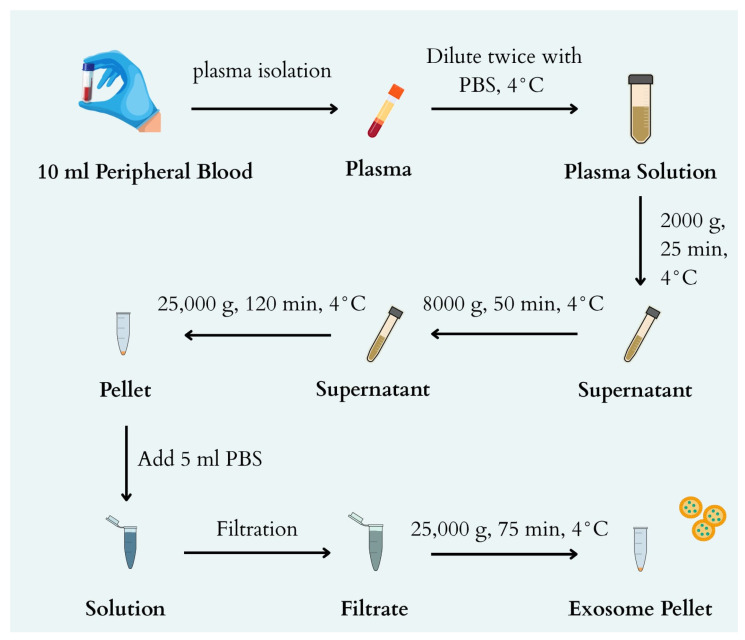
Diagram of the UC based exosome isolation process.

Prior to the UC process, the plasma was thawed on ice. After thawing and vortexing the plasma, the sample was transferred to a new 15 mL Falcon tube and diluted twice with cold 1× PBS (4 °C) without Mg2+ and Ca2+ ions (Figure 6) (Thermo Fisher Scientific, Waltham, MA, USA; cat. no. 14190144). The diluted plasma samples were centrifuged at 4 °C and 2000× *g* (OHAUS FRONTIER 5916R, Ohaus Corporation, Parsippany, NJ, USA) for 25 min. The supernatant was then transferred to an ultracentrifuge tube. The tubes containing the material were centrifuged in a tilt-rotor (Thermo Scientific S52-ST 2554, Thermo Fisher Scientific, Waltham, MA, USA) at 4 °C and 8000× *g* (Sorvall MX 150+, Thermo Fisher Scientific, Waltham, MA, USA) for 50 min. After centrifugation, the supernatant was transferred to a new ultracentrifuge tube. The tubes containing the material were centrifuged at 25,000× *g* for 120 min at 4 °C using a tilting rotor. After centrifugation, the supernatant was removed from the pellet (which may not be visible) and 5 mL of cold 1× PBS was added, and the entire mixture was mixed. The mixture was collected using a syringe with a needle. The needle was then removed, and a CA filter with a pore size of 0.22 μm (Merck Millipore) was attached to the syringe. The next step was to filter the mixture into a new ultracentrifuge tube. The tubes containing the filtrate were centrifuged at 25,000× *g* for 75 min at 4 °C using a tilting rotor. After centrifugation, the supernatant was removed from the pellet (which may not be visible), 100 μL of cold 1× PBS was added, and the entire mixture was mixed. The mixture was collected with an automatic pipette, transferred to a 1.5 mL Eppendorf tube, and then frozen at −80 °C.

### 4.4. Size Exclusion Chromatography

The stationary phase for SEC was Sepharose™ CL-2B (Sigma-Aldrich, St. Louis, MO, USA; CL2B300–500 ML), and the mobile phase was 1× PBS without Mg^2+^ and Ca^2+^ ions (Thermo Fisher Scientific, Waltham, MA, USA; cat. no. 14190144). 10 mL of Sepharose CL-2B were transferred to a 12 mL disposable polypropylene column (Bio-Rad Laboratories, Hercules, CA, USA; cat. no. 7321010) with a 30 μm polypropylene bottom filter and allowed to settle to form a stationary bed with an inner diameter of 15 mm and a height of 60 mm. The Sepharose CL-2B bed of the SEC column was washed with 10 mL of cold 1× PBS at room temperature to properly pack the column.

**Figure 7:**
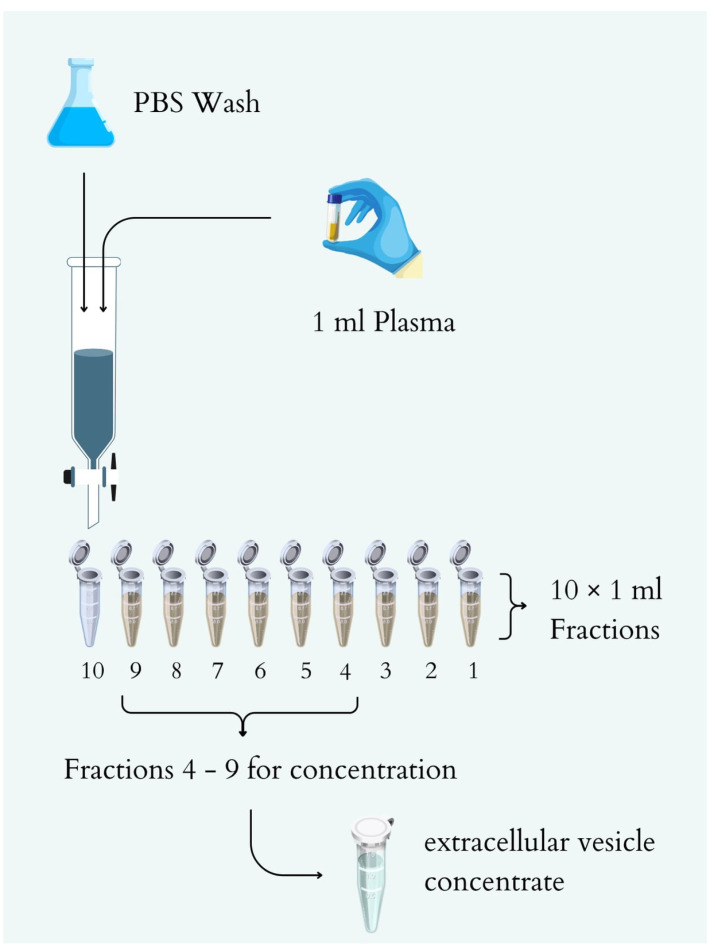
Diagram of the SEC -based exosome isolation process.

Before SEC, plasma was thawed on ice, vortexed, and applied to the column bed. Once the sample reached the top of the column, elution was carried out with 10 mL of cold 1× PBS without Mg^2+^ and Ca^2+^ ions, and the eluate was collected by gravity (Figure 7). From 1 mL of plasma, 10 fractions of 1 mL each were obtained. Protein concentration in each fraction was determined using a BCA protein assay kit (Thermo Scientific Pierce™, Thermo Fisher Scientific, Waltham, MA, USA) according to the manufacturer’s instructions. Fractions 4–9 were concentrated to obtain an extracellular vesicle concentrate using a VivaSpin500 concentrator with a 300 kDa cutoff (Sartorius, Göttingen, Germany). The resulting extracellular vesicle concentrate was transferred to a new Eppendorf tube and frozen at −80 °C.

After each chromatographic run, the SEC column was washed with 30 mL of 0.1 M NaOH solution (0.22 μm filtered), followed by 60 mL of 1× PBS without Mg2+ and Ca2+ ions, and then stored at 4 °C.

### 4.5. Commercial Exosome Isolation Kit

The procedure was performed using previously prepared plasma according to the manufacturer’s instructions (Exo-spin™ mini-HD kit, Cell Guidance Systems, Cambridge, United Kingdom; cat. no. EX05-20).

### 4.6. Protein Concentration Determination

The samples for measurement were prepared by adding 25 μL of protein lysis buffer to 25 μL of exosome preparation and then incubating at 50 °C for 10 min. The protein concentration in the exosome preparation was determined using a commercially available BCA kit according to the manufacturer’s instructions (Pierce™ BCA Protein Assay Kit, Thermo Fisher Scientific, Waltham, MA, USA; cat. no. A55864). Absorbance measurement was performed using a Multiskan SkyHigh Microplate Spectrophotometer (Thermo Fisher Scientific, Waltham, MA, USA).

### 4.7. Western Blot

Protein extraction was performed using 20 μL of the obtained exosome preparation and 20 μL of protein lysis buffer. The samples were loaded onto a 10% denaturing polyacrylamide gel and electrophoresed at 100 V for 3 h (Bio-Rad Mini Protean Tetra Cell, Bio-Rad Laboratories, Hercules, CA, USA) and then transferred to a PVDF membrane (C.B.S. Scientific EBU-102, C.B.S. Scientific Company, San Diego, CA, USA) at 20 V for 12 h. After the transfer was completed, the membrane was cut and blocked in a 5% skim milk solution for 1 h. The membrane was then incubated with the corresponding primary antibody for 2 h (Cell Signaling Technology, Danvers, MA, USA: CD9 (D8O1A) Rabbit mAb #13174, Alix (3A9) Mouse mAb #2171, CD81 (D3N2D) Rabbit mAb #56039, Calnexin Antibody #2433, Albumin Antibody #4929). After incubation, the membrane was washed 3 times with 1× TBS buffer and once with 1× TBST buffer. The membranes were then blocked with 5% BSA solution for 30 min. After blocking, they were incubated with the corresponding secondary antibodies (Cell Signaling Technology, Danvers, MA, USA: Anti-rabbit IgG #7074S, Anti-mouse #7076S7076S) for 1 h. The membranes were then washed 3 times with 1× TBS buffer and 1 time with 1× TBST buffer. The PVDF membranes with proteins were developed using a membrane imaging system (Chemidoc, Bio-Rad Laboratories, Hercules, CA, USA) with ECL chemiluminescent reagents (Clarity Western ECL Substrate, Bio-Rad Laboratories, Hercules, CA, USA).

### 4.8. Scanning Electron Microscope

To study exosomes samples were negatively stained on formvar/carbon-coated copper grids. The grids were floated on sample drops for 30 min, treated with 1% glutaraldehyde, washed in PBS and double-distilled water, and subsequently contrasted and dried using a mixture of 1.8% methyl cellulose and 0.3% uranyl acetate. The grids were then mounted on a specimen holder and sputter-coated with a thin gold/palladium layer prior to examination using a Zeiss Merlin VP Compact field-emission scanning electron microscope (Carl Zeiss, Oberkochen, Germany).

### 4.9. Enzyme-Linked Immunosorbent Assay

96-well plates were coated with 100 μL of monoclonal antibody CA125 [X75], CK19 [1H6], or TAG72/CA72.4 [B72.3] (Invitrogen, Thermo Fisher Scientific, Waltham, MA, USA) at a concentration of 3 μg/μL and incubated overnight at 4 °C. After blocking with 0.5% BSA and washing with PBST (1× PBS + 0.05% Tween-20), 100 μL of exosome suspension isolated from patient plasma was added and incubated for 3 h at room temperature. Subsequently, the same primary antibody was added again, diluted in 0.5% BSA/PBS, and incubated for 2 h at 4 °C. After washing, Goat anti-Mouse IgG (H + L) Superclonal™ HRP-conjugated secondary antibody (Invitrogen, Thermo Fisher Scientific, Waltham, MA, USA) was added and incubated for 1 h at room temperature. The reaction was developed using TMB substrate and absorbance was measured at 450 nm. HSP70 assay was performed using the commercial Human HSP70 ELISA Kit (Invitrogen, Thermo Fisher Scientific, Waltham, MA, USA), according to the manufacturer’s instructions. Absorbance measurement was performed using a Multiskan SkyHigh Microplate Spectrophotometer (Thermo Fisher Scientific, Waltham, MA, USA).

### 4.10. Statistical Analysis

Statistical analyses were performed using Statistica 13.1 software (StatSoft, Tulsa, OK, USA). The concentrations of HSP70, CK19, CA125, and TAG72 determined by ELISA were presented as means ± SEM (standard error of the mean) from two independent experiments. The distribution of variables was assessed using the Shapiro–Wilk test. Differences between groups were analyzed using the Mann–Whitney U test (non-parametric) or Student’s *t*-test (parametric), depending on the data distribution Values of *p* < 0.05 were considered statistically significant (* *p* < 0.05, ** *p* < 0.01, *** *p* < 0.001).

## Figures and Tables

**Table 1:** Protein levels in exosome preparations.

Method Used for Isolation	Protein Level (mg/mL)
Ultracentrifugation	0.919
Chromatography SEC	1.036
Exosome isolation kit	1.161

**Table 2:** Demographic characteristics of CRC patients and control subjects.

Characteristics	CRC Patients	Control Subjects
**Number, n**	40	40
**Sex, n (%)** Female Male	16 (40.0%) 24 (60.0%)	23 (57.5%) 17 (42.5%)
**Age, mean (±SD)** Female Male Overall	61.0 (9.2) 61.5 (10.2) 61.3 (9.7)	62.3 (5.6) 65.6 (5.6) 63.8 (5.8)

Data are presented as mean ± SD. Note: An additional 12 CRC patients were included only in the pilot methodological phase and were excluded from the statistical analyses.

## Data Availability

The original contributions presented in this study are included in the article. Further inquiries can be directed to the corresponding author.

## Abbreviations

The following abbreviations are used in this manuscript:

CRC	Colorectal cancer
UC	Ultracentrifugation
SEC	Size exclusion chromatography
ELISA	Enzyme linked immunosorbent assay
ESCRT	Endosomal sorting complex required for transport
ER	Endoplasmic reticulum
BCA	Bicinchoninic acid assay
DLS	Dynamic light scattering
CA	Cellulose acetate
SEM	Standard error of the mean
